# Feasibility of 3-dimensional printed models in simulated training and teaching of transcatheter aortic valve replacement

**DOI:** 10.1515/med-2024-0909

**Published:** 2024-02-28

**Authors:** Yu Mao, Yang Liu, Yanyan Ma, Mengen Zhai, Lanlan Li, Ping Jin, Jian Yang

**Affiliations:** Department of Cardiovascular Surgery, Xijing Hospital, Air Force Medical University, Xi’an, 710032, Shaanxi, China; Department of Cardiovascular Surgery, Xijing Hospital, Air Force Medical University, 127 Changle West Road, Xi’an, 710032, Shaanxi, China

**Keywords:** cardiovascular 3D printing, transcatheter aortic valve replacement, simulation, training

## Abstract

In the study of TAVR, 3-dimensional (3D) printed aortic root models and pulsatile simulators were used for simulation training and teaching before procedures. The study was carried out in the following three parts: (1) experts were selected and equally divided into the 3D-printed simulation group and the non-3D-printed simulation group to conduct four times of TAVR, respectively; (2) another 10 experts and 10 young proceduralists were selected to accomplish three times of TAVR simulations; (3) overall, all the doctors were organized to complete a specific questionnaire, to evaluate the training and teaching effect of 3D printed simulations. For the 3D-printed simulation group, six proceduralists had a less crossing-valve time (8.3 ± 2.1 min vs 11.8 ± 2.7 min, *P* < 0.001) and total operation time (102.7 ± 15.3 min vs 137.7 ± 15.4 min, *P* < 0.001). In addition, the results showed that the median crossing-valve time and the total time required were significantly reduced in both the expert group and the young proceduralist group (all *P*<0.001). The results of the questionnaire showed that 3D-printed simulation training could enhance the understanding of anatomical structure and improve technical skills. Overall, cardiovascular 3D printing may play an important role in assisting TAVR, which can shorten the operation time and reduce potential complications.

## 1 Introduction

Since Cribier et al. [1] conducted the first transcatheter aortic valve replacement (TAVR) in 2002, it has been increasingly popularized in clinical practice, and its indications have also been extended from patients who cannot tolerate surgeries to high- and intermediate-risk patients and now proceduralists may even operate in certain low-risk cases. After 20 years of development, a large amount of evidence-based research has reinforced the idea that TAVR, as a leading and revolutionary technology, is changing the treatment strategy and direction of aortic valve diseases, which leads to less trauma, lower risk, faster recovery speed, and better prognosis in patients [2,3,4].

However, the difference between TAVR and surgical aortic valve replacement (SAVR) lies in the fact that it is difficult to look directly at the full view of the aortic root, let alone to open the heart to observe the internal anatomic structures [5]. However, computed tomography (CT), magnetic resonance imaging, echocardiography, and other examinations may only provide a 2-dimensional field of view, and proceduralists still need to carefully study and measure this field before surgery [6]. Therefore, TAVR accelerates the in-depth integration of imaging, engineering, materials, interventional medicine, surgery, and other disciplines and presents new challenges for the formulation of precise and personalized TAVR strategies for patients to reduce potential complications and improve long-term prognosis.

With the continuous development of medical visualization, digital modeling is required to be increasingly accurate, and the requirement of precision is particularly important in cardiovascular diseases [7]. The emergence of cardiovascular 3-dimensional (3D) printing has brought new ideas and methods to many complex diagnoses and treatments of cardiovascular diseases, provided proceduralists with individualized models, and helped them be clear about the anatomic structures at a glance [8,9,10]. With the continuous improvement 3D printing, there have been scattered reports that this technology may assist the successful implementation of TAVR [11,12,13]. The preprocedural computed tomography angiography (CTA) data of patients may be used for 3D reconstruction and printing into an aortic root model, providing more intuitive and visual information that is difficult to see in traditional imaging methods [14,15]. 3D printing combined with TAVR allows proceduralists to improve doctor–patient communication and to train young proceduralists and medical students [12,16]. In addition, 3D-printed models may be used to simulate conditions *in vitro* to assess the possibility of coronary artery obstruction, paravalvular leakage, conduction block, and other serious complications and to develop personalized preprocedural plans and help improve the safety and efficacy of procedures [17,18].

Simulation is an important part of medical teaching. Some have already been available for a variety of applications, especially for training purposes. Studies have shown the importance of practical participation in stimulating the interest of medical students; this trend is driven by the desire to improve the quality of patient care and to ensure safety [19,20,21,22,23]. Therefore, this study intends to explore the training role of using 3D-printed aortic root models and pulsatile simulators to conduct simulation training to assist TAVR to improve the success rate and reduce potential complications.

## 2 Methods

### 2.1 The process of 3D-printing aortic root models

**Figure 1:**
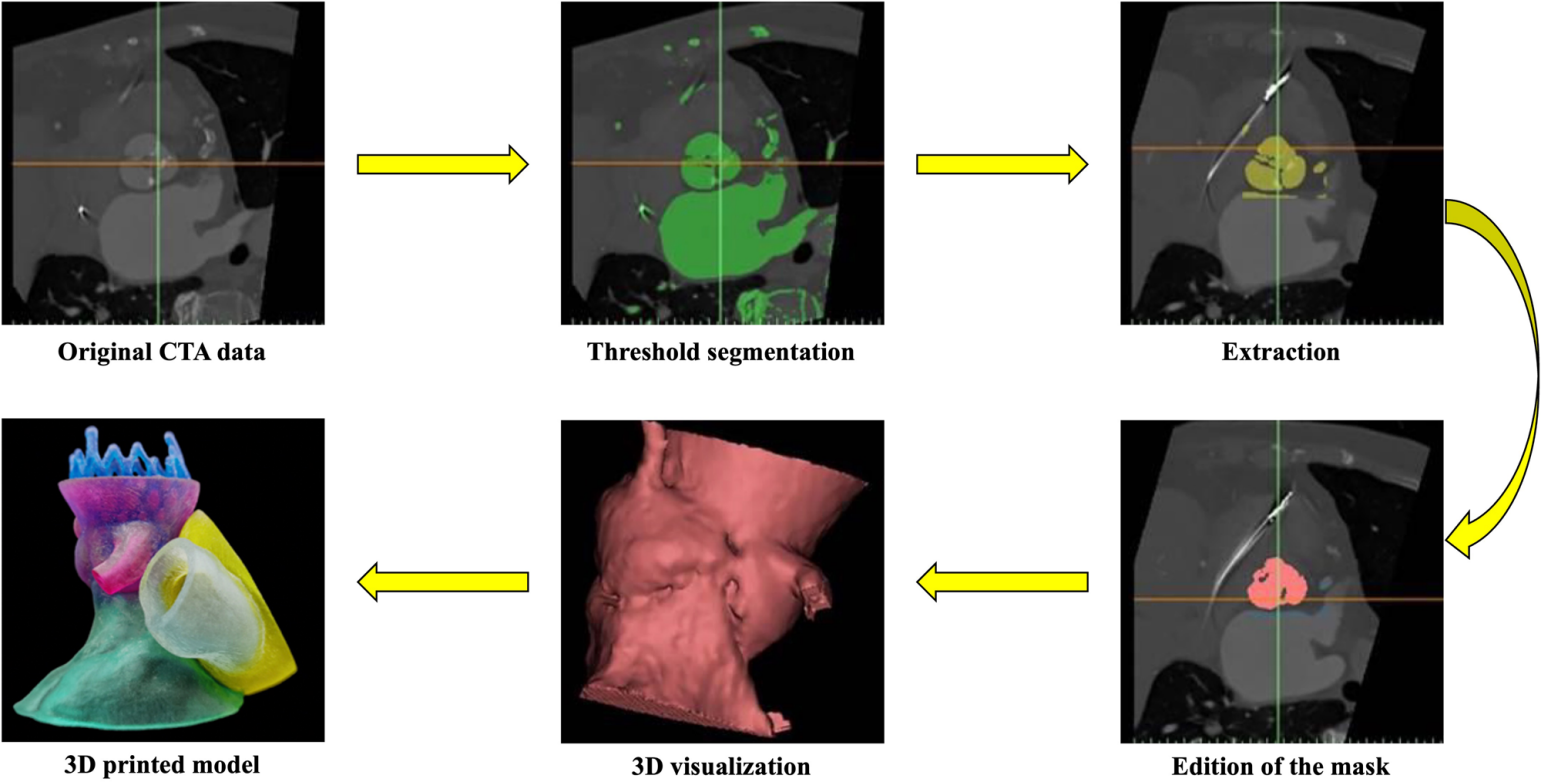
The main process of 3D-printed aortic root models.

The patient’s CTA data were imported into Materialise Mimics version 21.0 (Leuven, Belgium) software, and the threshold segmentation function was used to segment the 3D reconstruction. Then, the obtained 3D reconstructive model was extracted, trimmed, smoothed, and repaired digitally in the Materialise 3-Matic (Leuven, Belgium) software. The structure of the aortic root was restored completely. The standard tessellation language (STL) files of the 3D reconstruction were exported to a Stratasys Polyjet 850 multimaterial full-color 3D printer. Different tissues of the aortic root were printed with materials of different hardnesses and colors to obtain the patient’s 3D-printed aortic root model (Figure 1).

### 2.2 Pulsatile simulators

**Figure 2:**
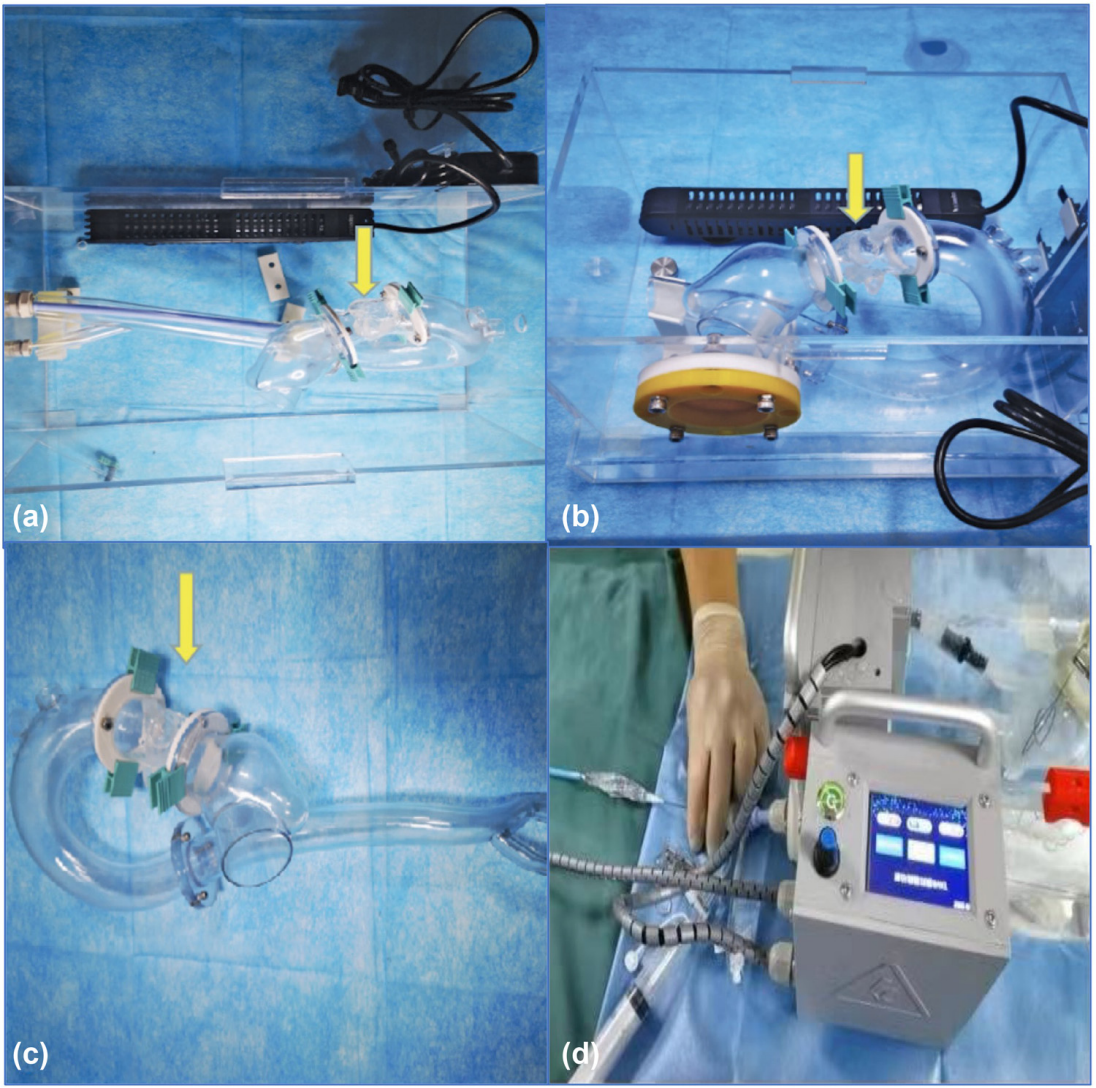
The 3D-printed aortic root models and pulsatile simulators. (a) The pulsatile simulators for TAVR via the peripheral artery approach. (b) The pulsatile simulators for TAVR via the apex approach. (c) The 3D-printed aorta model used for crossing-valve simulation. (d) The driving segment of the TAVR pulsatile simulator. Yellow arrows indicate the detachable 3D-printed aortic root model. 3D, 3-dimensional; TAVR, transcatheter aortic valve replacement.

First, the patient’s CT data were divided into the ventricle, the aortic root, and the aortic arch to measure the inner diameters and the corresponding radians. According to the measured data, specific engineers print the corresponding parts of the 3D reconstructed models and design the buckles so that each part is convoluted closely (Figure 2a and b). The simulator mainly comprises two segments: (1) the working segment includes the transfemoral artery/transapical approach and the 3D printed aortic root model and (2) the driving segment includes the circulating pump, the complete connection loop, and the control system (Figure 2c and d). By setting up the pulsatile simulator (pressured pulsatile blood flow), the 3D-printed aortic root model and adjacent tissues may simulate movements under a real pathophysiological state. At the same time, high-speed cameras, X-ray equipment, and other related instruments were used for measurement and evaluation to obtain various data similar to the clinical data, including important indicators such as the hemodynamic characteristics of the aortic valve.

### 2.3 Guidance for 3D printing

**Figure 3:**
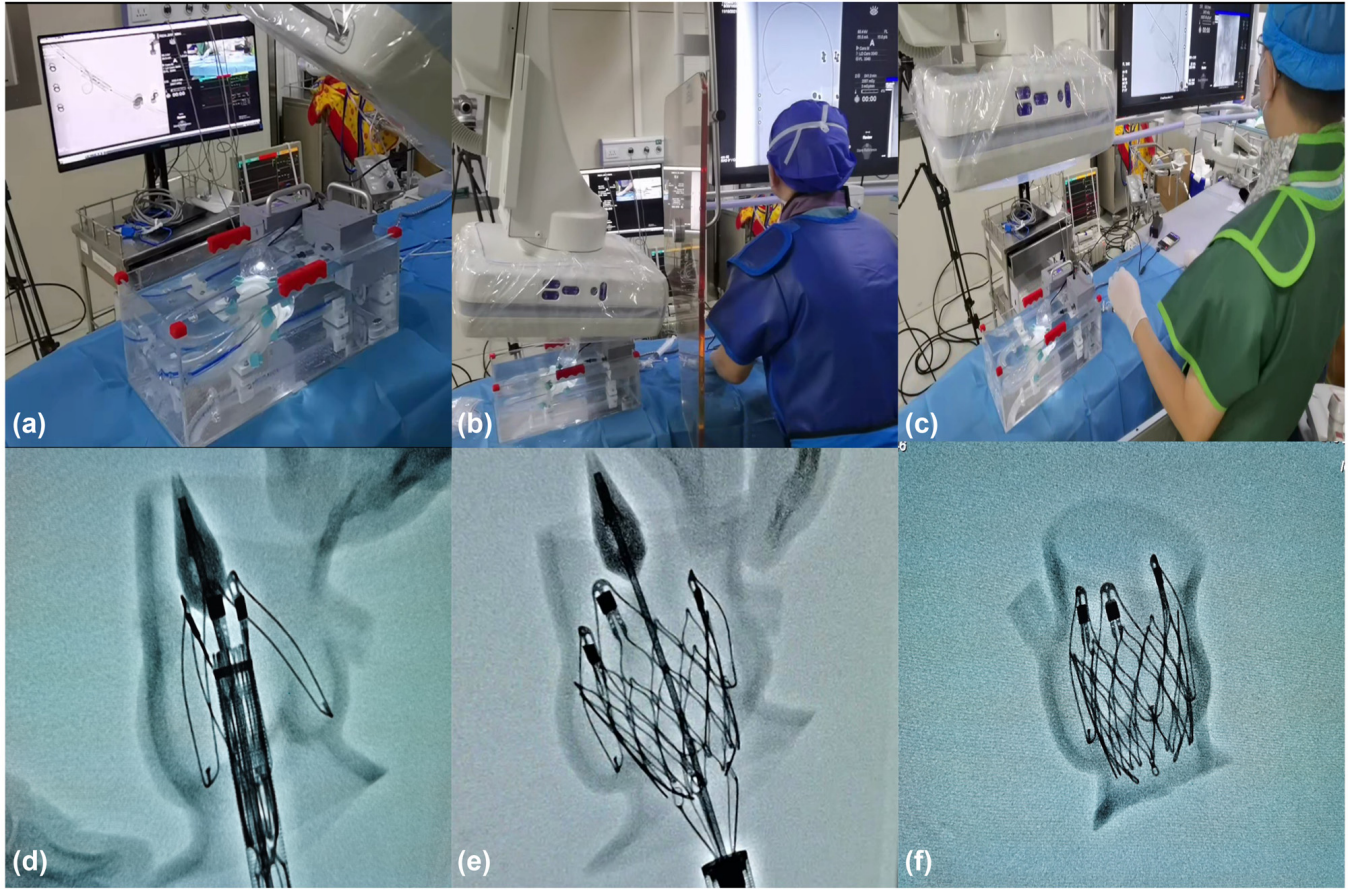
Simulation training using 3D-printed aortic root models and pulsatile simulators. (a and b) The simulation using different balloon sizes to observe the changes in leaflets and calcification. (c) Preprocedural simulations using the 3D-printed aortic root model to predict paravalvular leakage. (d) Preprocedural simulations using the 3D-printed aortic root model to predict conduct blockage. (e) Trainees simulated TAVR using the pulsatile simulator. (f) Balloon expansion was simulated using a pulsatile simulator via a peripheral artery approach. 3D, 3-dimensional; TAVR, transcatheter aortic valve replacement.

**Figure 4:**
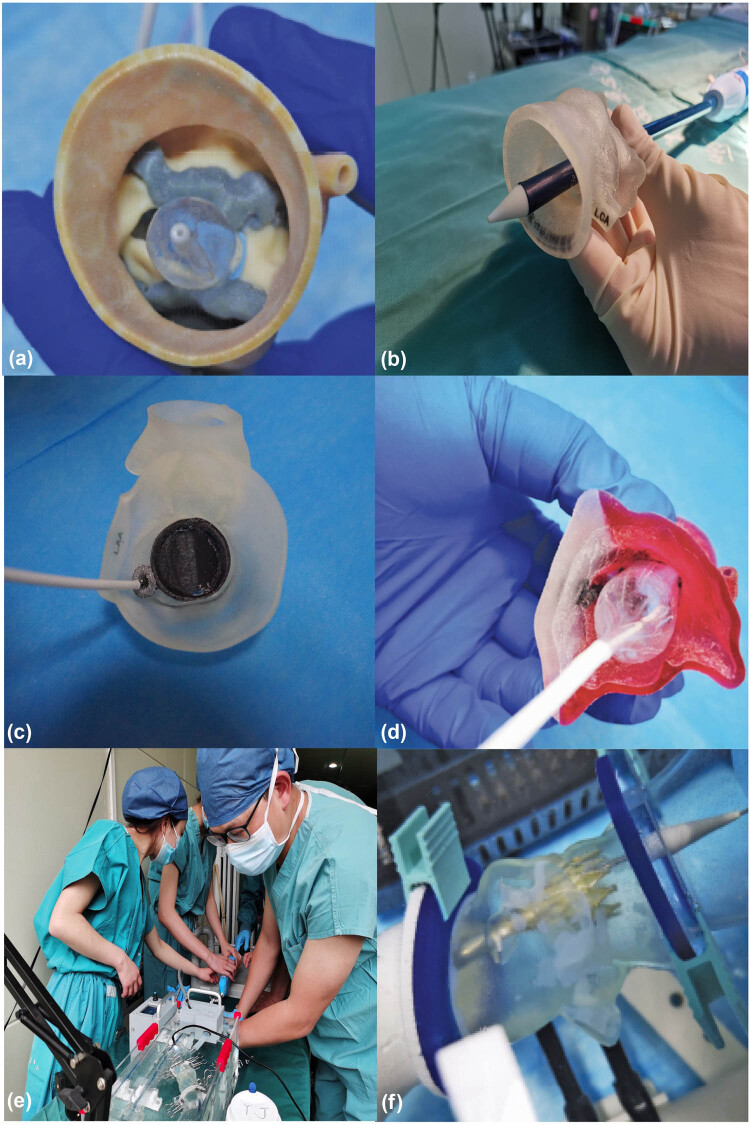
Simulation training on the bench test. (a) The pulsatile simulator was prepared for TAVR simulation. (b) The proceduralist was training the procedural step of crossing the valve. (c) The proceduralist was training the procedural step of stent release. (d) The stent was positioned precisely in the DSA image. (e) The stent was released in the DSA image. (f) After the stent was released, the DSA image showed that the stent attached well to the 3D-printed model. TAVR, transcatheter aortic valve replacement; DSA, digital subtracted angiography; 3D, 3-dimensional.

Simulation training is very important for the professional growth of young cardiovascular specialists. With the rapid development of computer graphics, bioengineering, and digital modeling, TAVR training systems based on 3D printing will bring more simulation training opportunities. Trainees may practice several main procedural steps (such as crossing the valve, exchanging the thread, and positioning the stent) by using the specific 3D printed model to enhance their procedural skills and proficiency to improve the learning curve (Figure 3). According to the types of aortic valves, different products, and stents were selected for simulation to evaluate the risks of paravalvular leakage, conduction block, coronary artery blockage, and vascular complications (Figure 4).

### 2.4 The 3D-printed simulation group versus the non-3D-printed simulation group

**Figure 5:**
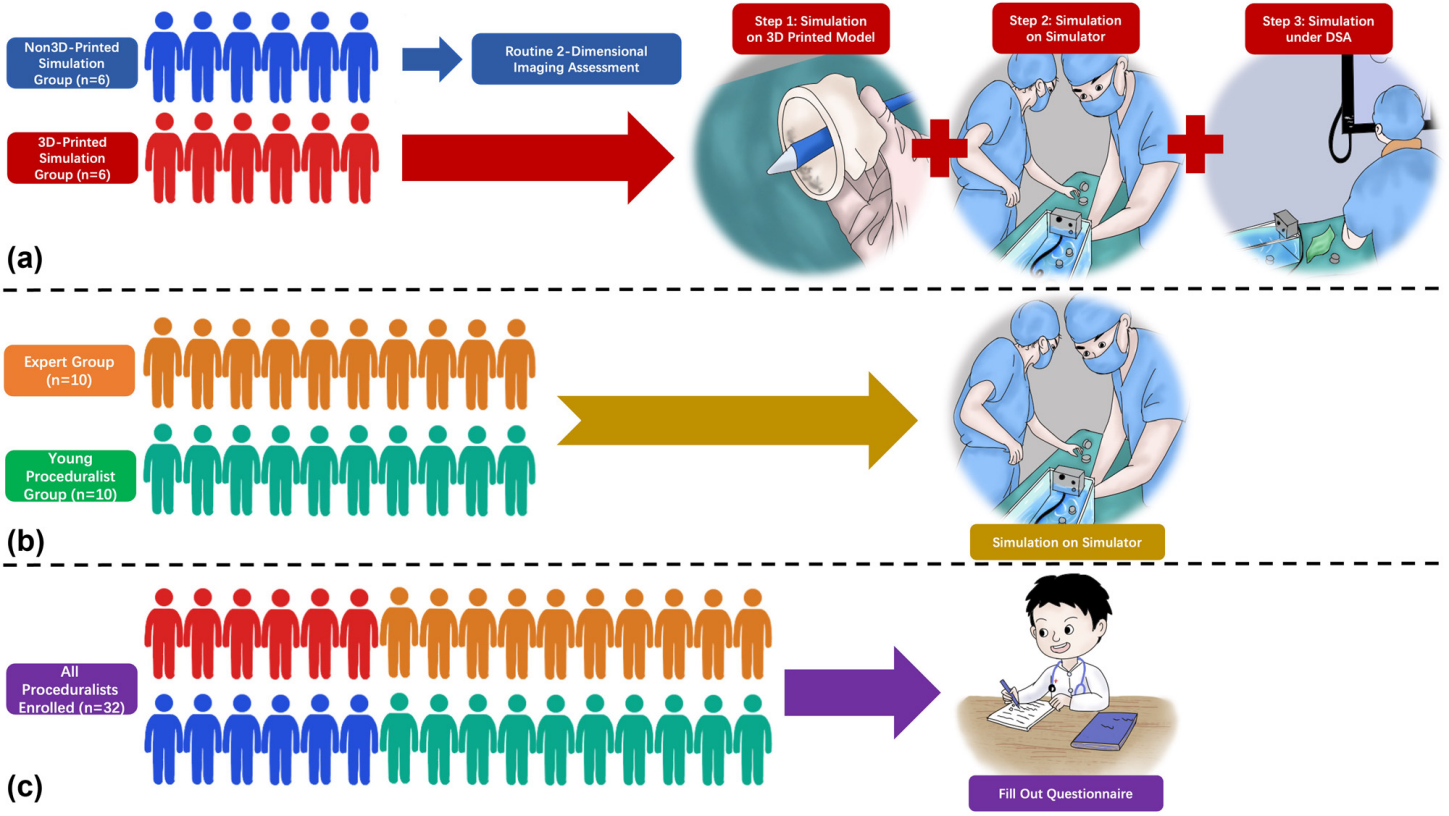
The flowchart of this study. (a) The 6 proceduralists in non-3D-printed simulation group conducted routine 2-dimensional imaging assessment before TAVR and the 6 proceduralists in 3D-printed simulation group completed three steps of simulations successively before TAVR. (b) The expert group (*n* = 10) and the young proceduralist group (*n* = 10) both completed 3 times of simulations on the pulsatile simulator. (c) All 32 proceduralists filled out the questionnaire.

The flowchart of the whole study is shown in Figure 5. All 48 patients with aortic regurgitation were divided into the 3D-printed simulation group and the non-3D-printed simulation group, with 24 patients in each group. Twelve experts were selected and equally divided into two groups. Six proceduralists in the 3D-printed simulation group had simulated procedural steps on the bench test before TAVR, and the proceduralists in the non-3D-printed simulation group carried out TAVR with conventional preoperative evaluation (Figure 5a). Each expert completed four operations, and the crossing-valve time, the total time, and occurrence of major postprocedural complications were recorded and analyzed statistically.

### 2.5 The expert group versus the young proceduralist group

Besides, another 10 experts and 10 young proceduralists were selected, which were divided into the expert group and the young proceduralist group, with 10 members in each group. Each expert or young proceduralist received the simulation three times and recorded the crossing-valve time and the total simulation time (Figure 5b). The results of the first, second, and third simulation sessions were compared in pairs and analyzed statistically.

### 2.6 Questionnaire

Overall, all above 32 doctors were organized to complete a specific questionnaire, to evaluate the training and teaching effect of 3D-printed simulations (Figure 5c). The questionnaire mainly included the following three aspects: the overall perspective, the simulation steps, and the model materials. A collection of completed questionnaire results, a comprehensive understanding of anatomical structures of the aortic root, procedural steps of TAVR, and the application of various devices with the guidance of 3D printing simulation were obtained. The details are shown in Table 1.

**Table 1:** Questionnaire for evaluation of specific training by 3D printing

Items	Rating				
**The Overall Perspective**					
Better understand the aortic root anatomical structures and procedural locations	1	2	3	4	5
Improve the confidence to complete TAVR	1	2	3	4	5
Training may lead to increased patient safety	1	2	3	4	5
Shorten TAVR learning curve	1	2	3	4	5
**The Simulation Steps**					
Practice the steps of the surgery	1	2	3	4	5
Use various types of guidewires and catheters skillfully	1	2	3	4	5
Help make the choice of stent graft diameters	1	2	3	4	5
Help make the choice of the releasing depth	1	2	3	4	5
Identify difficulties and improve technical skills	1	2	3	4	5
**The Model Material**					
Provide the necessary information about the relevant major pathological findings	1	2	3	4	5
Consistency and elasticity similar to that of the human myocardium	1	2	3	4	5
Authenticity and applicability of the simulation and measurement	1	2	3	4	5

Note. Ratings are on a Likert scale: 1 (not at all) to 5 (very much).

### 2.7 Statistical analysis

For continuous variables, the results were summarized with the number of observations and the mean ± standard deviation (SD), the median (minimum, maximum) for hospital length of stay, and 95% confidence interval by normal approximation. Quantitative discrete and categorical ordinal variables are displayed as medians and interquartile ranges (IQRs; 25th–75th percentiles) and were analyzed with the non-parametric test. A two-tailed *P* value of <0.05 was considered statistically significant. All statistical analyses were conducted by using Statistical Package for Social Sciences (SPSS, Chicago, Illinois, USA) version 25.0.


**Clinical trial registration:** ClinicalTrials.gov Protocol Registration System (NCT02917980).
**Consent to participate:** The patients/participants provided their written informed consent to participate in this study.
**Consent for publication:** Written informed consent was obtained from the individual(s) for the publication of any potentially identifiable images or data included in this article.
**Ethics approval:** The studies involving human participants were reviewed and approved by Clinicaltrials Organization: Xijing Hospital, Air Force Medical University.

## 3 Results

### 3.1 The 3D-printed simulation group versus the non-3D-printed simulation group

**Figure 6:**
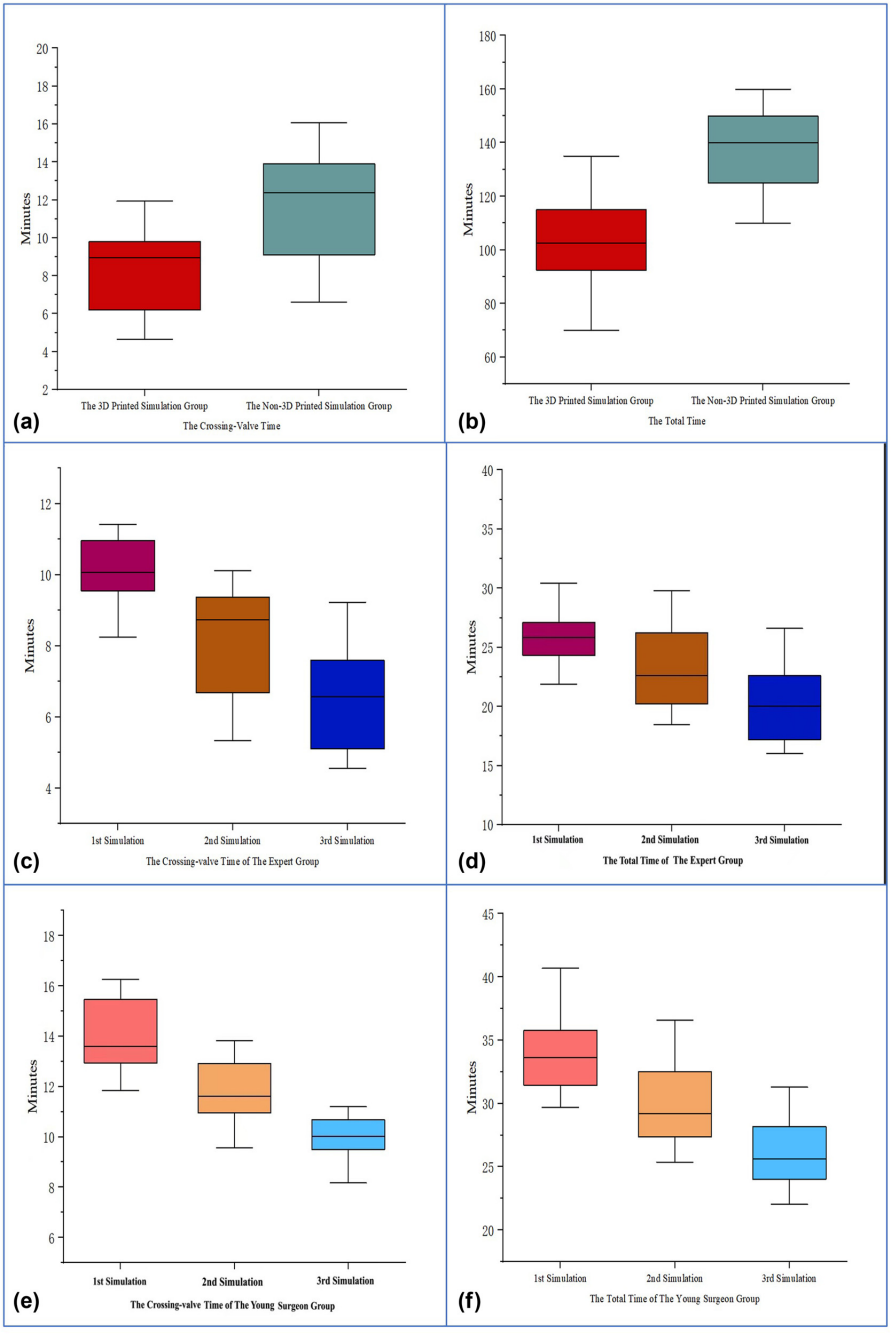
The outcomes of TAVR simulations. (a) The comparison of crossing-valve time between the 3D-printed simulation group and the non-3D-printed simulation group (*P* < 0.001). (b) Comparison of the total time between the 3D-printed simulation group and the non-3D-printed simulation group (*P* < 0.001). (c) The comparison of crossing-valve time between three simulations in the expert group (*P* < 0.001). (d) The comparison of total time between three simulations in the expert group (*P* < 0.001). (e) The comparison of crossing-valve time between three simulations in the young proceduralist group (*P* < 0.001). (f) The comparison of total time between three simulations in the young proceduralist group (*P* < 0.001). *P* value from paired Student’s *t* test. TAVR, transcatheter aortic valve replacement; 3D, 3-dimensional.

Forty-eight patients with aortic regurgitation were selected in Xijing Hospital. A total of 27 males and 21 females were included, and the mean age was 68.8 ± 6.1 years. The baseline information of 48 patients is shown in Table 2. In the 3D-printed simulation group, the mean age was 41.3 ± 2.9 years old, and the mean working experience was 13.0 ± 2.5 and 9.7 ± 1.6 years for interventions. In the non-3D-printed simulation group, the mean age was 41.8 ± 2.6 years, and the mean working experience was 12.8 ± 2.3 and 9.7 ± 1.5 years for interventions. The baseline information of the experts is shown in Table 3. All 12 experts successfully completed the four operations, and the intraoperative data are shown in Table 4. For the non-3D-printed simulation group, six proceduralists required a crossing-valve time of 11.8 ± 2.7 min and a total time of 137.7 ± 15.4 min. In contrast, six proceduralists of the 3D-printed simulation group decreased the crossing-valve time to 8.3 ± 2.1 min and decreased the total time to 102.7 ± 15.3 min (Figure 6a and b). In addition, no major postprocedural complications (such as coronary artery obstruction and conduction block) occurred in the 3D-printed simulation group. Only one patient developed a mild paravalvular leakage after the operation, which was much better than that in the non-3D-printed simulation group (1/24 vs 5/24, *P* = 0.046).

**Table 2:** Baseline characteristics of patients (*n* = 48)

Characteristics	
Age (years)	68.8 ± 6.1
Male	27 (56.3)
Height (cm)	168.2 ± 8.3
Weight (kg)	68.4 ± 7.7
Body mass index (kg/m^2^)	24.6 ± 4.8
Systolic pressure (mm Hg)	132.8 ± 14.5
Diastolic pressure (mm Hg)	72.9 ± 10.2
NYHA class III or IV	31 (64.6)
STS score (%)	6.276 (2.620–11.956)
EuroSCORE II (%)	3.0 (0.8–11.2)
Comorbidities	
Diabetes	13 (27.1)
Coronary artery disease	4 (8.3)
Anemia	2 (4.2)
Dyslipidemia or hyperlipidemia	26 (54.2)
Chronic obstructive pulmonary disease	10 (20.9)
PPM/ICD	1 (2.1)
Pulmonary hypertension*	5 (10.4)
Chronic kidney disease†	9 (18.8)
Severe liver disease‡	1 (2.1)
Prior gastrointestinal hemorrhage	1 (2.1)
Prior stroke/TIA	6 (12.5)
Malignancy	3 (6.3)
Previous cardiac intervention	
CABG	4 (8.3)
PCI	3 (6.3)
Electrocardiogram	
Atrial fibrillation	15 (31.3)
RBBB	7 (14.6)
LBBB	8 (16.7)

Values are presented as *n* (%) or median (25th, 75th percentile). *mPAP ≥ 25 mm Hg. †Defined as eGFR < 60 mL/min. ‡Defined as MELD-albumin score >12. ICD, implantable cardioverter defibrillator; STS, Society of Thoracic Surgeons; EuroSCORE, European system for cardiac operative risk evaluation; NYHA, New York Heart Association; PPM, permanent pacemaker; TIA, transient ischemic attack; CABG, coronary artery bypass grafting; PCI, percutaneous coronary intervention; RBBB, right bundle branch block; LBBB, left bundle branch block.

**Table 3:** Baseline information of experts in the 3D-printed simulation group and the non-3D-printed simulation group (*n* = 12)

Number	Sex	Age (years)	Years for proceduralist	Years for intervention
A1	Male	43	12	8
A2	Male	40	11	8
A3	Male	46	17	12
A4	Male	39	12	10
A5	Male	42	15	11
A6	Male	38	11	9
B1	Male	45	16	10
B2	Male	39	10	8
B3	Male	44	15	12
B4	Male	41	13	10
B5	Male	43	12	10
B6	Male	39	11	8

A group represents the 3D-printed simulation group; B group represents the non-3D-printed simulation group.

**Table 4:** Results of the 3D-printed simulation group versus the non-3D-printed simulation group

Group	1st operation		2nd operation		3rd operation		4th operation		*P* value (crossing valve)	*P* value (total)
Time	Crossing valve	Total	Crossing valve	Total	Crossing valve	Total	Crossing valve	Total		
A1	6′44″	80′00″	5′09″	90′00″	5′23″	120′00″	4′39″	105′00″	3.2 × 10^−4^	0.051
B1	7′13″	160′00″	8′42″	135′00″	9′32″	130′00″	8′45″	150′00″		
A2	5′18″	100′00″	6′31″	100′00″	5′51″	70′00″	5′27″	115′00″	2.3 × 10^−4^	0.009
B2	9′21″	110′00″	6′37″	155′00″	8′11″	160′00″	8′52″	145′00″		
A3	9′37″	120′00″	8′23″	100′00″	8′18″	95′00″	7′49″	105′00″	2.1 × 10^−4^	0.018
B3	13′13″	135′00″	12′28″	150′00″	12′10″	140′00″	11′08″	140′00″		
A4	10′50″	110′00″	10′07″	100′00″	9′13″	115′00″	9′44″	85′00″	2.5 × 10^−4^	0.021
B4	14′32″	145′00″	13′41″	135′00″	13′18″	145′00″	12′59″	140′00″		
A5	11′56″	95′00″	9′33″	110′00″	8′48″	90′ 00″	9′29″	135′00″	1.2 × 10^−4^	0.041
B5	14′31″	155′00″	15′17″	155′00″	12′19″	130′00″	12′38″	125′00″		
A6	9′05″	120′00″	9′53″	80′00″	10′46″	85′00″	10′24″	100′00″	2.6 × 10^−4^	0.006
B6	16′04″	125′00″	14′51″	145′00″	14′08″	140′00″	12′17″	150′00″		

Group A represents the 3D-printed simulation group; group B represents the non-3D-printed simulation group.

### 3.2 The expert group versus the young proceduralist group

The baseline information of the proceduralists is shown in Table 5. In the expert group, the mean age was 42.5 ± 2.3 years, and the mean working experience was 12.5 ± 2.1 and 10.0 ± 2.1 years for interventions. In the young proceduralist group, the mean age was 30.6 ± 1.7 years, and the mean working experience was 4.2 ± 1.5 and 2.0 ± 0.9 years for interventions. Ten experts and ten young proceduralists successfully completed three simulations. The time required for procedures is shown in Table 6. For the expert group, the median crossing-valve time and the total time required in the first simulation were 10.0 ± 1.1 and 26.4 ± 3.5 min, respectively. In the second simulation and the third simulation, the median crossing-valve time was reduced to 8.1 ± 1.6 and 6.5 ± 1.5 min, and the total time was reduced to 23.3 ± 3.8 and 20.3 ± 3.5 min, respectively. For the young proceduralist group, the median crossing-valve time and the total time required in the first simulation were 14.0 ± 1.5 and 33.9 ± 3.3 min, respectively. In the second simulation and the third simulation, the median crossing-valve time was reduced to 11.8 ± 1.3 and 9.7 ± 1.4 min, respectively, and the total time was reduced to 29.9 ± 3.5 and 26.2 ± 3.1 min, respectively (Figure 6c–f). The comparisons of time consumed by the 20 proceduralists during each simulation are shown in Table 7. From the results above, we observed that the median crossing-valve time and the total time required were significantly reduced from the first to the third simulation. The reduced time was not associated with the trainees’ level of experience.

**Table 5:** Baseline information of proceduralists in the expert group and the young proceduralist group (*n* = 20)

Number	Sex	Age (years)	Years for proceduralist	Years for intervention
C1	Male	41	11	9
C2	Male	42	13	10
C3	Male	39	10	7
C4	Male	44	13	11
C5	Male	46	15	11
C6	Male	43	16	14
C7	Male	41	10	8
C8	Male	45	14	12
C9	Male	44	12	9
C10	Male	40	11	9
D1	Male	31	3	2
D2	Male	33	4	2
D3	Male	29	2	1
D4	Male	30	5	2
D5	Male	28	4	1
D6	Male	31	4	2
D7	Male	32	6	4
D8	Male	30	3	2
D9	Male	33	7	3
D10	Male	29	4	1

C group represents the expert group; D group represents the young proceduralist group.

**Table 6:** Results of the expert group versus the young proceduralist group

Rank	1st		2nd		3rd	
	Device	Total	Device	Total	Device	Total
C1	9′33″	24′19″	6′41″	20′15″	5′06″	17′47″
C2	8′15″	21′53″	5′20″	18′29″	4′33″	16′01″
C3	10′13″	26′40″	8′50″	22′26″	8′08″	20′33″
C4	9′49″	26′11″	9′22″	24′55″	7′36″	19′29″
C5	11′04″	30′26″	9′28″	27′57″	7′12″	24′44″
C6	9′55″	25′30″	7′11”	22′46″	6′23″	20′40″
C7	11′25″	33′45″	10′07″	29′48″	9′14″	26′36″
C8	8′24″	22′43″	6′13″	19′10″	5′39″	17′11″
C9	10′42″	25′41″	8′38″	21′18″	4′49″	16′52″
C10	10′58″	27′07″	9′06″	26′13″	6′45″	22′38″
D1	14′27″	35′47″	13′11″	32′30″	11′12″	28′09″
D2	13′40″	31′26″	10′59″	29′14″	9′55″	24′53″
D3	11′51″	29′42″	9′34″	25′22″	6′29″	22′02″
D4	16′16″	40′41″	13′50″	36′35″	10′06″	31′17″
D5	13′31″	33′59″	10′57″	29′08″	9′34″	26′45″
D6	15′28″	35′16″	11′48″	31′13″	9′30″	26′22″
D7	12′56″	30′19″	11′25″	27′21″	10′34″	23′18″
D8	13′31″	31′47″	12′30″	26′26″	11′54″	24′00″
D9	12′25″	33′15″	10′48″	28′10″	8′10″	24′36″
D10	15′49″	36′21″	12′55″	33′27″	10′41″	30′45″

The C group represents the expert group; the D group represents the young proceduralist group.

**Table 7:** Comparisons of time consumed by the 20 proceduralists during each simulation

*P* values from comparison tests between simulations		
**C group**	**Crossing valve**	**Total**
1st vs 2nd simulation	2.8 × 10^−5^	2.3 × 10^−5^
2nd vs 3rd simulation	0.0009	1.5 × 10^−5^
1st vs 3rd simulation	8.0 × 10^−6^	7.0 × 10^−8^
**D group**	**Crossing valve**	**Total**
1st vs 2nd simulation	1.5 × 10^−5^	8.5 × 10^−7^
2nd vs 3rd simulation	0.0002	1.0 × 10^−6^
1st vs 3rd simulation	1.1 × 10^−5^	5.3 × 10^−9^

The C group represents the expert group; the D group represents the young proceduralist group.

### 3.3 Questionnaire

First, in The Overall Perspective, all the trainees considered that 3D-printed simulation is helpful for understanding the anatomical structures of the aortic root and could shorten the learning curve and improve confidence to complete surgery. Moreover, in The Simulation Steps, the trainees deemed that the simulation could deepen the understanding of procedures, identify technical difficulties, and improve technical skills. In particular, it is of great help in the skillful use of guide wires and catheters, the choice of stent graft diameter, and the release depth. Finally, in terms of The Model Materials, the trainees recognized that the model could provide necessary information about the main pathological findings and the consistency and elasticity of the materials with the human myocardium and considered that the simulation and measurement were authentic and applicable (Table 8).

**Table 8:** Results of the questionnaire

Items	Mean ± SD
**The Overall Perspective**	
Better understand the aortic root anatomical structures and procedural locations	4.9 ± 0.3
Improve the confidence to complete TAVR	4.8 ± 0.5
Training may lead to increased patient safety	4.2 ± 0.8
Shorten TAVR learning curve	5.0 ± 0.0
**The Simulation Steps**	
Practice the steps of the surgery	5.0 ± 0.0
Use various types of guidewires and catheters skillfully	4.6 ± 0.6
Help make the choice of stent graft diameters	4.6 ± 0.6
Help make the choice of the releasing depth	4.7 ± 0.5
Identify difficulties and improve technical skills	4.8 ± 0.4
**The Model Material**	
Provide the necessary information about the relevant major pathological findings	5.0 ± 0.0
Consistency and elasticity similar to that of the human myocardium	4.3 ± 0.8
Authenticity and applicability of the simulation and measurement	4.4 ± 0.6

Note. Ratings are on a Likert scale: 1 (not at all) to 5 (very much).

## 4 Discussion

Over the past 20 years, the rapid development of TAVR has provided new ideas and experience for minimally invasive treatments, and an increasing number of elderly patients with severe aortic valvular disease have been treated successfully [24,25]. Meanwhile, cardiovascular 3D printing has achieved great progress, with composite materials (different hardnesses of silicone, resin, polyethylene, and rubber) being used to print the aortic root model, which may effectively simulate the elastic structures of valves and the rigid structures of calcification to achieve a highly accurate anatomical reduction. Studies have shown that, on the basis of 2D images, 3D-printed aortic root models may visualize anatomic structures and increase the understanding of leaflet morphology, the distribution of calcification, and the distributions of sinuses and outflows [26,27,28,29,30]. In addition, the 3D-printed aortic root model may be used for predicting surgical risks, such as conduction block, paravalvular leakage, annulus rupture, and coronary artery blockage. The construction of a TAVR simulation platform is of great help to improve the training quality for and technical skills of young doctors [31]. However, current studies on simulation training lack sufficient evidence.

Studies have shown that the benefits of simulation-based training have been validated by a large meta-analysis, which indicated that simulation training may reliably influence outcomes related to trainees’ knowledge and skills. Moreover, patient-related outcomes seem to be influenced by simulation-based teaching [18]. The results of this study showed that, in terms of operation time, crossing-valve time, DSA time, radiation dose, and complications, the 3D-printed simulation group was significantly improved compared with the non-3D-printed simulation group. In addition, this study carried out comparison research based on simulation training and found that along with the increase in simulation times, the simulation proficiency of experts and young proceduralists increased, and the time required was significantly shortened. In conclusion, this study shows that 3D-printed simulation is widely applied in the perioperative evaluation of TAVR and plays a corresponding auxiliary guiding role in the development of procedural technology. In the process of individualized and precise intervention, the 3D-printed simulation will become another pair of eyes for proceduralists, which may reduce the operation time and the DSA time and effectively improve the success rate of surgery [14,32,33,34,35]. The simulation has an advantage in acquiring knowledge, and enhancing practical experience through training activities may have an impact on shortening the learning curve, which is necessary in TAVR teaching and training programs.

With the rapid development of imaging and 3D printing, 3D-printed models have been able to accurately formulate the anatomical structures of patients, to bring proceduralists visual information *in vitro*, and have been applied well in complex congenital heart disease, valvular disease, and other aspects. The personalized model may improve communication between doctors and patients, and proceduralists and medical students may also simulate procedures *in vitro*. It might not only train doctors and medical students but also make personalized preprocedural plans for patients, which plays an important guiding role in improving the success rate and safety of surgery. In the current situation, the properties of polymer materials used in 3D printing are still difficult to combine well with the elasticity and toughness, which limits the prediction accuracy of some complications in simulation. However, with the continuous improvement in safety requirements, it is believed that research on materials will also be vigorously promoted. In addition, in recent years, 3D bioprinting has made gratifying progress. With the development of biological engineering, materials, biology, computer science, and other multidisciplinary cooperation, although we still have a gap between the status quo and the clinical application, cardiovascular 3D printing for simulation training and teaching will continue to achieve new breakthroughs.

### 4.1 Limitations

Cardiovascular 3D-printed models play a unique role in the diagnosis and treatment of cardiovascular diseases due to their intuitive characteristics. However, the current application still has some limitations: (1) the accuracy needs to be strengthened. The data are derived from images (including CTA, magnetic resonance, and echocardiography). These examinations have their own limitations, which affect the recognition of subtle structures. (2) The materials need to be improved. The materials require the combination of various soft and hard materials and multiperformance materials, and the materials that can be used in cardiovascular models are limited at present. (3) The high cost. The production of the personalized model requires multidisciplinary collaboration to reflect the changes as much as possible. However, it takes a longer time to accomplish the product, and a higher price limits its wide application.

## 5 Conclusion

As an important application field of cardiovascular 3D printing, simulation has become a critical part of precision medicine. Patient-specific models may effectively formulate preprocedural plans and help reduce radiation exposure and surgical risks. The 3D-printed models applied in training may enhance doctors’ understanding of the anatomic structures and pathophysiological characteristics of cardiac diseases, improve their proficiency in procedures, enhance the effect of training, shorten the learning curve, and enhance practical experience.
